# Bicarbonate of Soda Dressings for Burns

**Published:** 1878-04

**Authors:** 


### Bicarbonate-of-Soda Dressing for Burns.

Dr. Ely McClelland, U. S. A., reports the following interesting cases in the *Louisville Medical News*. He says he has had considerable experience in the use of bicarbonate, and selects these as of the most interest:

Case I.—A half-breed Nez Perces child received a terrible scald of the first magnitude, involving the greater portion of the scalp, the right side of the face, the neck, shoulder, and arm of the same side. The wounded surface was covered with lint which had been soaked in a saturated solution of sodæ bicarbonatis, and was kept wet by constant applications of the same solution. The relief from pain was instantaneous. No slough occurred, and the child has recovered, saved from any cicatricial deformity.

Case II.—Act. Ass't Surg. Pring, U. S. A., in medical charge of the troops at Mt. Idaho, an outpost of this command, reports the following: The wife of an officer of the Second U. S. Infantry, who had accompanied her husband to the cantonments, from inability to obtain servants in that exposed locality, was herself engaged in preparing the early meal. Being inexperienced in such work, this lady poured water into a vessel containing boiling lard, and in the explosion which followed was severely scalded about the face and neck, involving the right eye. The bicarbonate-of-soda dressing was employed with the most decided benefit. The pain was instantly relieved, and no disfigurement resulted beyond the total loss of vision in the injured eye.

To secure successful results from this treatment it is necessary that the application be made of a saturated solution. A half pound of the bicarbonate should be added to a quart of water, and should be subjected to violent agitation. A sheet of patent lint of old linen sufficiently large to envelop the wounded surface should be thoroughly saturated with the solution, and the surface should be completely covered therewith; the dressing should never be permitted to become dry, but the solution should be freely and constantly used. No other dressing is necessary, but the lint first applied should not be disturbed for several days.

